# Habitual Intakes, Food Sources and Excretions of Phosphorus and Calcium in Three German Study Collectives

**DOI:** 10.3390/nu10020171

**Published:** 2018-02-02

**Authors:** Ulrike Trautvetter, Bianka Ditscheid, Gerhard Jahreis, Michael Glei

**Affiliations:** 1 Institute of Nutrition, Friedrich Schiller University Jena, Dornburger Straße 24, 07743 Jena, Germany; Bianka.Ditscheid@med.uni-jena.de (B.D.); Gerhard.Jahreis@uni-jena.de (G.J.); Michael.Glei@uni-jena.de (M.G.); 2 Institute of General Practice and Family Medicine, Jena University Hospital, Bachstraße 18, 07743 Jena, Germany

**Keywords:** phosphorus intake, calcium intake, human intervention study, food sources phosphorus, 24 h urine, weighed dietary record

## Abstract

Phosphorus intake in Europe is far above recommendations. We present baseline data from three human intervention studies between 2006 and 2014 regarding intake and excretion of phosphorus and calcium. All subjects documented their nutritional habits in weighed dietary records. Fasting blood samples were drawn, and feces and urine were quantitatively collected. Dietary phosphorus intake was estimated based on weighed dietary records and urine phosphorus excretions. Food sources were identified by allocation to defined food product groups. Average phosphorus consumption was 1338 mg/day and did not change from 2006 to 2014, while calcium intake decreased during this period (1150 to 895 mg/day). The main sources for phosphorus intake were bread/cereal products, milk/milk products and meat/meat products/sausage products and the main sources of calcium intake included milk/milk products/cheese, bread/cereal products and beverages. There was no difference between estimated phosphorus intake from the weighed dietary records and urine phosphorus excretion. In conclusion, we demonstrated constant phosphorus intakes far above the recommendations and decreasing calcium intakes below the recommendations in three German collectives from 2006 to 2014. Furthermore, we could show in case of usual intakes that an estimated phosphorus intake from urine phosphorus excretion is similar to the calculated intake from weighed dietary records.

## 1. Introduction

In Europe and other Western societies, the average dietary phosphorus intake is far above recommendations [1,2]. This is mainly a result of increased consumption of food prepared or treated with phosphate additives, which range from baked goods to cola beverages [3]. Phosphorus itself plays an essential role in cellular biology (e.g., nucleic acid synthesis, energy metabolism, enzyme activity), thus it is present in every body fluid [4]. Under these circumstances it is not surprising that an imbalance of phosphorus metabolism may lead to several clinical diseases, for example rhabdomyolysis (hypophosphatemia), acute nephropathy and renal failure (hyperphosphatemia) [5]. Hyperphosphatemia is discussed very critically, and consequently, the role of dietary phosphorus intake, too [6,7]. Under normal physiological conditions, the amount of phosphorus absorbed in the gut is equivalent to the phosphorus excreted by the kidney. The postprandial increase of plasma/serum phosphate concentration leads to an increased renal phosphorus excretion and to a subsequent normalization of plasma phosphate concentration. Some hormones and factors are involved in this process e.g., fibroblast growth factor 23 (FGF23) and parathyroid hormone (PTH). FGF23 regulates high plasma phosphate concentrations by increasing renal phosphorus excretion and decreasing phosphorus absorption in the gut, while PTH inhibits renal phosphorus reabsorption resulting in a decrease in plasma phosphate concentration [8]. Phosphorus and calcium metabolism are closely linked as they share regulation factors such as Vitamin D and PTH, and both minerals represent the main components of bone mass (hydroxyapatite). In fact, a chronic imbalance of dietary calcium and phosphorus intake leads to bone loss [9]. Long-term plasma phosphate concentrations in the upper limit of normal are associated with mortality and cardiovascular diseases in healthy subjects and in patients with chronic kidney disease (CKD) [10,11]. Yet, the role of dietary phosphorus intake is still not clear [12,13].

Over the past 8 years we performed a number of human intervention studies with different phosphate and calcium compounds. This publication presents baseline data from three studies investigating habitual intake and excretion of phosphorus and calcium (assessed by weighed dietary records) that are so far not published. The objectives were to (I) compare phosphorus and calcium intakes from three different time points (2006, 2011 and 2014); (II) determine the main food sources of phosphorus and calcium (2014) and (III) compare a calculation method for the phosphorus intake based on 24 h urine phosphorus concentrations with the phosphorus intake estimated from weighed dietary records (2006, 2011, 2014).

## 2. Materials and Methods

### 2.1. Study Design and Subjects

**Figure 1:**
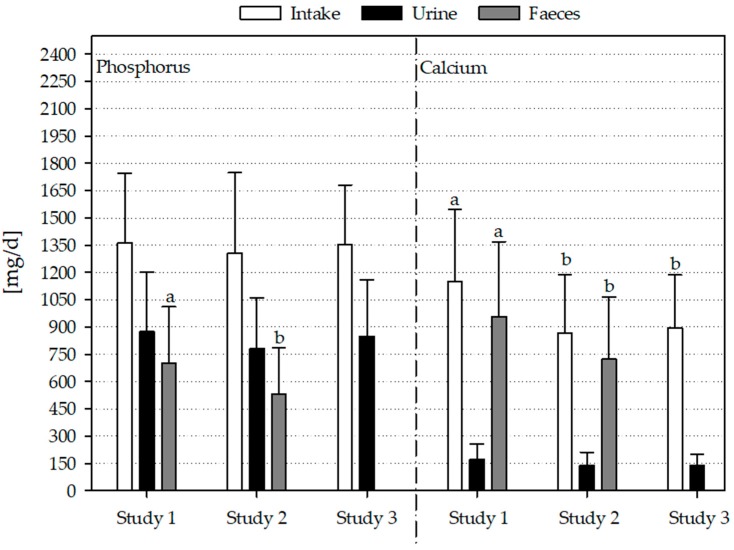
Habitual intake, renal and fecal excretion of phosphorus and calcium in three studies; *n* = 31 (Study 1); *n* = 56 (Study 2); *n* = 62 (Study 3); data are expressed as mean + standard deviation; ^a,b^ dissimilar superscript letters indicate significant differences between the studies; results without superscript letters are not significantly different; differences between the studies were tested with univariate analysis of variance followed by Bonferroni post hoc test.

In this publication baseline data of three human intervention studies were analyzed (Figure 1). A concise overview over the studies’ characteristics is outlined in Table 1. Results and further information from Study 2 and 3 are published elsewhere (Study 2 [14], Study 3 [15]). Results of Study 1 were not published yet.

Study 1 and 2 were conducted at the Department of Nutritional Physiology of the Friedrich Schiller University Jena from October 2006–March 2007 and from January–April 2011, respectively. Study 3 was conducted at the Department of Nutritional Toxicology of the Friedrich Schiller University Jena between March and July 2014.

The three studies were performed according to the guidelines of the Declaration of Helsinki and all procedures involving human subjects were approved by the Ethical Committee of the Friedrich Schiller University of Jena (Study 1: 1828-07/06; Study 2: 2959-11/10; Study 3: 3987-01/14). Written informed consent was obtained from all subjects. The trials were registered at ClinicalTrials.gov (Study 1: retrospectively as NCT03286673; Study 2: NCT01297023; Study 3: NCT02095392).

Exclusion criteria for all three studies included diseases of the gastrointestinal tract, pregnancy, nursing and intake of any medication (e.g., for thyroid gland) or dietary supplements. In Study 3, additional exclusion criteria included post-menopausal age of women and subjects with baseline glomerular filtration rates below 80 mL/min/1.73 m^2^.

The results represent baseline data from 149 subjects of phosphorus and calcium intakes, plasma/serum concentrations and excretions in urine and feces. All subjects documented their normal nutritional habits in a dietary record for 3 (Study 1 and 2) or 7 days (Study 3), respectively. The subjects were encouraged to weigh all eaten foods with provided scales. All subjects collected their urine quantitatively (Study 1: 3 days; Study 2 and 3: 1 day). In addition, subjects collected their feces (Study 1 and 2: quantitatively for 3 days, Study 3: 1 feces sample). Fasting blood samples were drawn from all subjects.

The study was completed by 31, 56 and 62 subjects in Study 1, 2 and 3, respectively. Baseline characteristics including age, body weight and BMI are presented in Table 2.

### 2.2. Sample Preparation

Subjects and samples from each subject were coded to protect volunteer identity and to mask treatment groups during sample collection and analysis.

Blood samples were drawn by venipuncture and collected in serum and lithium heparin tubes. All tubes were centrifuged and the supernatants were stored at −80 °C until analysis (potassium EDTA 2500× *g*, 10 min, 20 °C; lithium heparin 4302× *g*, 7 min, 20 °C; serum: 2500× *g*, 10 min, 20 °C).

Fecal samples of Study 1 and 2 were immediately transported to the study center. Each specimen was weighed, frozen and stored at −20 °C. At the end of the studies, fecal samples were homogenized and portioned. The collected 24 h urine was transported to the study center after each day of collection in Study 1 and at the day of the blood sampling in Study 2 and 3. The urine volume from every participant was measured and aliquots were frozen at −20 °C until analysis.

### 2.3. Food Analysis

The intakes of phosphorus and calcium from the weighed dietary records were verified using the Prodi^®^ software (Nutri-Science GmbH, Freiburg, Germany; versions 4.5 (Study 1), 5.4, (Study 2) and 5.9 (Study 3)). All food items from the weighed dietary records of Study 3 were listed according to the amount consumed and with the respective phosphorus and calcium contents (version 6.6; Prodi^®^ software, Nutri-Science GmbH, Freiburg, Germany). Afterwards, single food items were allocated to product groups according to the “National Consumption Study II” [16]. Food sources of phosphorus and calcium (as percent) were calculated from phosphorus and calcium contents and the consumed amount of each food item. Examples for food items are listed in Table S1.

### 2.4. Blood Analysis

The analysis of serum minerals for Study 1 was performed on the autoanalyser Synchron LX or CX (Beckman Coulter, Brea, CA, USA) and on the autoanalyser ARCHITECT C16000 (Abbott, Chicago, IL, USA) for Study 2 and 3 according to the manufacturer’s recommendations at the Institute of Clinical Chemistry and Laboratory Medicine, Jena University Hospital.

### 2.5. Faeces and Urine Analysis

Phosphorus and calcium concentrations in feces and urine of Study 1 were determined after pressure digestion using ICP-OES (Optima 3000, Perkin Elmer, Waltham, MA, USA).

In Study 2 and 3 the concentrations of urinary phosphorus and calcium were measured according to certified methods of the Institute of Clinical Chemistry and Laboratory Medicine, Jena University Hospital on the autoanalyser ARCHITECT C16000 (Abbott, Chicago, IL, USA) [15]. Fecal phosphorus and calcium concentrations were determined after acid treatment by ICP-OES (iCAP 6000 ICP Spectrometer, Thermo Scientific, Waltham, MA, USA) [15].

### 2.6. Calculation of the Phosphorus Intake from 24 h Urine

The estimated phosphorus intake from 24 h urine was calculated according to Morimoto et al. (2014): estimated phosphorus intake from 24 h urine [mg/d] = urine phosphorus excretion [mg/day]/0.65 [17].

### 2.7. Statistics

Data analyses were performed using the statistical software package IBM SPSS Statistics 24 (SPSS Inc. IBM Company, Chicago, IL, USA). Variance homogeneity was tested using the Levene test.

Univariate analysis of variance followed by Bonferroni post hoc test was used to compare study results between the studies. In case of variance heterogeneity univariate analysis of variance with Welch adjustment followed by Tamhame-T2 post hoc test was done.

To compare the estimated phosphorus intake from 24 h urine with the intake calculated from the weighed dietary records Student’s t test (in case of variance homogeneity) and Wilcoxon signed-rank test (in case of variance heterogeneity) were used.

For correlation analysis, data were tested for normal distribution with the Kolmogorov-Smirnoff-Test. The Pearson correlation coefficient was used in case of normal distribution, and the Spearman correlation coefficient was used in case of rejection of normal distribution.

Differences were considered significant at *p*-level < 0.05.

## 3. Results

### 3.1. Habitual Intake and Excretion of Phosphorus and Calcium

The study collectives had significantly different ages, whereby subjects of Study 1 were the youngest and those of Study 2 were the oldest (Table 2). The subjects body weight and BMI were comparable between the studies.

Phosphorus intake, fecal and renal excretion as well as concentration of phosphate in plasma/serum of all studies are summarized in Figure 1 and Table 3 (for separate results of women and men see Table S2). Subjects of Study 2 showed the lowest plasma/serum phosphate concentration, followed by those of Study 3 and Study 1 (all subjects and women, Table 3, Table S1). Men of Study 1 had a significantly higher plasma/serum phosphate concentration compared to the male subjects of Study 2 (Table S2).

Fecal phosphorus excretion of all subjects was significantly higher in Study 1 compared to Study 2 (Figure 1; Study 3: no analysis of fecal excretion). Considering women and men separately, there was no difference in fecal phosphorus excretion (Table S2).

The intake of calcium differed between the three studies with significantly higher intakes in Study 1 compared to Study 2 and 3. The separated consideration of women and men did not show a significant difference between the three studies.

Serum calcium concentration was significantly higher in Study 1 compared to plasma calcium concentrations in Study 2 and 3. The same results were observed considering women and men separately.

Fecal calcium excretion was significantly lower in Study 2 compared to Study 1. This was due to significantly lower fecal calcium excretion of men from Study 2 compared to those from Study 1.

The phosphate concentrations in plasma/serum showed a significant inverse correlation with age in all subjects and gender-specific in men (all subjects: *r* = −0.505; *p* ≤ 0.001; men: *r* = −0.483, *p* ≤ 0.001; women: *r* = −0.029, *p* > 0.05). Phosphorus intake of all subjects showed significant positive correlations with calcium intake and with renal and fecal phosphorus and calcium excretion (Table 4). Calcium intake was significantly positively correlated with phosphorus and calcium excretion in urine and feces. In the case of women, phosphorus intake was significantly positively correlated with calcium intake, and fecal calcium and phosphorus excretion, whereas a significant inverse correlation was found for calcium plasma/serum concentration. Furthermore, calcium intake was significantly positively correlated with phosphorus intake and with phosphorus and calcium excretion in feces. With regard to men, phosphorus intake was significantly positively correlated with calcium intake and renal phosphorus excretion. Calcium intake was significantly positively correlated with phosphorus intake and fecal phosphorus excretion as well as with fecal and renal calcium excretion. With regard to all subjects and to men calcium intake was significantly inversely correlated with age.

### 3.2. Phosphorus and Calcium Sources of Study 3

**Figure 2:**
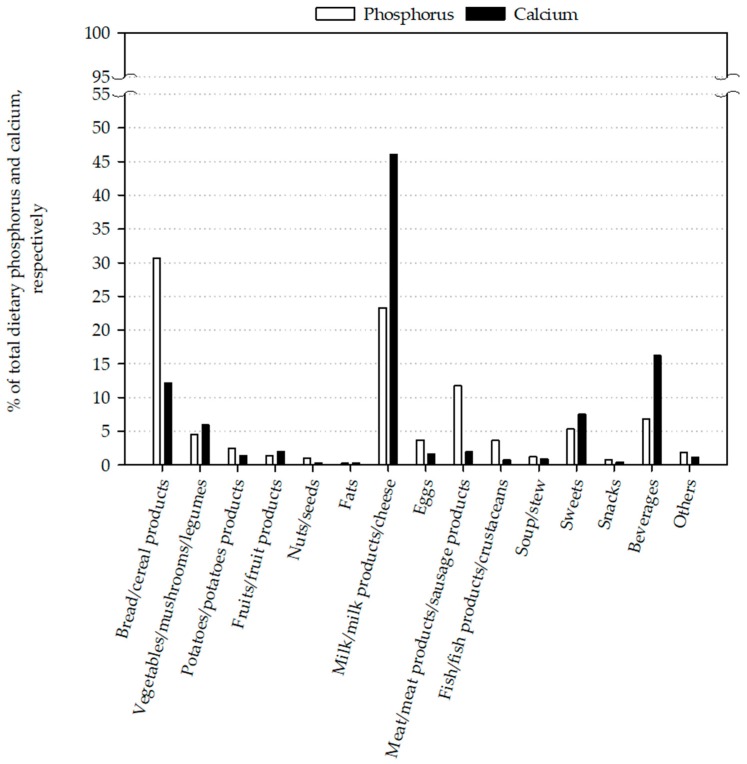
Major food sources for phosphorus and calcium of study 3.

The three main food product groups responsible for the phosphorus intake were bread/cereal products (30.7%), milk/milk products/cheese (23.3%) and meat/meat products/sausage products (11.7%) (Figure 2). Bread was the main source of phosphorus intake at 17.5%, followed by cheese/curd with 12.9%. Milk/milk beverages were responsible for 6.8% of the phosphorus intake (Table 5).

Milk/milk products/cheese (46.0%), beverages (16.2%) and bread/cereal products (12.1%) were the main sources of calcium intake (Figure 2). Cheese/curd (24.8%) was the main calcium source, followed by milk/milk beverages (13.0%), water (7.6%) and bread (6.1%) (Table 5).

### 3.3. Comparisons of Calculation Methods of Phosphorus Intake

With regard to all subjects, there were no significant differences between phosphorus intake calculated from weighed dietary records and estimated phosphorus intake from 24 h urine (Table 6).

In the case of women, phosphorus intake (weighed dietary records; 1188 ± 295 mg/day) was significantly higher compared to estimated phosphorus intake from 24 h urine (1032 ± 303 mg/day). Similar differences between the two calculation methods exist for all studies considered separately, however only in Study 3 were differences significant (Table S2).

The estimated phosphorus intake (urine) showed a significant positive correlation to phosphorus intake (weighed dietary records) in all subjects and in men (Spearman correlation coefficient; all subjects: *r* = 0.411, *p* ≤ 0.001; men: *r* = 0.334, *p* ≤ 0.01).

## 4. Discussion

Phosphate additives are nearly ubiquitously distributed in today’s food supply [18]. According to Calvo and Uribarri (2013), phosphate additives incorporated during processing can result in an additional phosphorus intake of 500 mg/day. In the United States, mean phosphorus intake was 1399 mg/day in 2014 [19]. In Europe, phosphorus intake ranged from 1000 to 2313 mg/day as estimated by the European Food Safety Authority (EFSA) [2]. In a Spanish population (18–64 years) mean phosphorus intake was 1175 mg/day and in a German population (mean age: 41 years) phosphorus intake was 1327 mg/day [20,21]. The EFSA reported for adult populations (18–65 years) the highest phosphorus intake in France, Italy and the Netherlands with approximately 2000–2300 mg/day [2]. In fact, these intakes are far above the recommendations. The Institute of Medicine (Bethesda, MD, USA) and the German Association for Nutrition recommend a daily phosphorus intake of 700 mg/day [22,23]. Furthermore, the EFSA defines 550 mg phosphorus/day as an adequate intake for adults [2]. Unfortunately, data about current habitual phosphorus intakes of German populations are scarce and thus, it is useful to publish results of small sample sizes, too.

The present study showed a mean phosphorus intake of approximately 1350 mg/day. The investigation period (2006 to 2014) had no significant influence on the habitual phosphorus intake. McClure et al. (2017) published phosphorus intake data from the National Health and Nutrition Examination Survey (NHANES) in the United States from 2001 to 2014. These data included phosphorus intakes of 34,000 US adults and the results showed no increase in phosphorus intake (2001: 1373 mg/day, 2014: 1399 mg/day) [19]. Considering men and women, it is worth mentioning that men consumed more than twice as much phosphorus as recommended (approx. 1500 mg/day phosphorus). This is in accordance with NHANES and the latest EFSA report and likely caused by the larger quantities of food consumed per day by men [2,19].

Dietary phosphorus intake consists of two sources: organic and inorganic phosphate [24]. Phosphorus from organic phosphates is part of animal and vegetarian protein present in meat, milk, legumes and nuts [25]. The intestinal absorption rate of organic phosphorus varies between 40% and 60% [24]. Phosphorus from inorganic phosphates derives mainly from food additives, such as ortho-, mono-, di-, tri- or poly-phosphates [25]. The use of phosphorus additives is widespread ranging from baked goods to cola beverages and serves to regulate acidity, emulsify or preserve food [3]. This added phosphorus is not protein bound, thus is well absorbable in the intestinal tract (>90%) [25]. In Study 3, the main sources of dietary phosphorus were bread and cheese/curd followed by meat and meat products. These results are in accordance with the results of the NHANES 2001–2014, in which bread/cereal products (29.3%) were the main source of phosphorus intake, followed by meat/poultry/fish/mixtures (25.2%) and milk/milk products (21.0%) [19]. These product groups are rich in protein, thus rich in phosphorus [25]. Unfortunately, we were not able to identify the amount of added phosphorus in the different product groups, however it is known that processed foods contain phosphate additives such as baked goods, sausages, and soft drinks [3]. These three product groups alone amounted to 11.2% of the ingested phosphorus in Study 3. Given that phosphorus intake is highly correlated with calcium intake (*r* = 0.597, *p* ≤ 0.001), it is not astonishing that the main sources of calcium in Study 3 were milk/milk products/cheese (46.0%) and bread/cereal products (12.1%). Therefore, a shift from a dairy-rich to a dairy-free diet leads not only to a decreased calcium intake of 1100 mg/d, but also to a reduced phosphorus intake of 900 mg/day [26]. Furthermore, reducing the intake of naturally phosphorus-rich foods could lead to a decreased protein intake and maybe to protein energy malnutrition [27]. This suggests that a conscious reduction of phosphorus intake should not only be achieved by a reduced intake of naturally phosphorus-rich foods, such as milk and meat. To reduce a very high phosphorus intake, foods and product groups with a high amount of phosphate additives should be avoided or consumed moderately. These products are mainly processed foods, such as “fast food”, convenience products, sausages, soft drinks, baked goods and fully pre-made meals [3]. Given that these products are associated with metabolic syndrome (which is associated with an increased risk of cardiovascular events) they should be consumed in moderation anyway [28,29,30].

Currently available data demonstrate that habitual phosphorus intake is far above the recommendations. Takeda et al. (2014) reported that long-term high phosphorus intake leads to negative effects on bone health [9]. Other publications stated that a high phosphorus intake is associated with increased risk of cardiovascular diseases and of mortality [12,31,32].

The results of our three studies revealed no correlation between phosphorus intake and phosphate concentrations in serum/plasma. Furthermore, supplemented phosphorus (as calcium phosphate and phosphate) did not increase plasma phosphate concentrations. This is due to the fact that in healthy subjects, the kidney is the main excretion organ of phosphorus and in states of high phosphorus intake, renal phosphorus excretion increases [8,15]. However, kidney diseases are often characterized by an impaired glomerular filtration rate (GFR). In the first stages of CKD the phosphorus homeostasis (especially the phosphate concentration in plasma) is maintained by PTH and FGF23, but when GFR continues to fall (GFR < 50 mL/min) compensatory mechanisms fail leading to hyperphosphatemia [33,34]. Hyperphosphatemia is strongly associated with increased cardiovascular morbidity and mortality in healthy subjects and in patients with CKD [10,35]. It is known that phosphate homeostasis is impaired very early in the course of CKD and that patients with moderate CKD (but with normal phosphate concentrations in plasma) have an increased mortality risk [36,37]. Presently, CKD is underdiagnosed and undertreated [38]; therefore, it may be possible that a high phosphorus diet increases the individual risk. In Study 3, GFR were determined with the Chronic Kidney Disease Epidemiology Collaboration equation for estimating the glomerular filtration rate (CKD-EPI). Due to exclusion criteria of Study 3, all subjects with GFR under 80 mL/min/1.73 m^2^ were excluded and thus, the kidney function of this study collective was not impaired. However, age is significantly inversely correlated with plasma/serum concentration of phosphate in all subjects as well as in gender specific analysis in men. This is in accordance with the fact, that Study 2 with the oldest subjects showed the lowest phosphate concentrations in plasma/serum. Cirillo et al. (2010) stated that serum phosphate concentration progressively declines with aging, because of a parallel decline in phosphate reabsorption of the renal proximal tubule. This effect was shown for both sexes [39]. However, in women the authors observed a transient increase of serum phosphate concentration during the perimenopausal period [39,40]. This fact could explain why there was no correlation detectable between plasma/serum phosphate concentration and age by the women in our studies.

However, an adequate calcium intake could counteract the negative effects of high phosphorus intakes [9,41]. A diet high in phosphorus and calcium leads to an increase in fecal phosphorus and calcium excretion, which could allude to the formation of amorphous calcium phosphate in the intestine [14,15,42,43]. By forming amorphous calcium phosphate, phosphorus absorption decreases potentially explaining the constant plasma concentration after high phosphorus diets [14,15]. This assumption is supported by the positive correlation between phosphorus intake and fecal phosphorus and calcium excretion in our studies—even without supplementation. However, habitual calcium intake in Study 1 was above and in Study 2 and 3 it was below the recommended calcium intake of 1000 mg/day [22,23]. Therefore, it is worth noting that an imbalance between phosphorus and calcium intake could also lead to disturbances of calcium absorption due to the formation of amorphous calcium phosphate [15].

The main limitation of our and other comparable studies is that intake data are a result of calculations. While NHANES calculated dietary intake based on 24 h recalls, our studies used weighed dietary records for calculation. Despite the different sources of dietary input (24 h recall and weighed dietary record) NHANES and our studies have one important similarity: all calculations are based on databases with phosphorus contents of different foods [19]. According to the literature, these databases have inaccuracies in phosphorus contents because of regional and seasonal variation in naturally contained phosphorus. Moreover, the additional phosphorus from the widespread and varying use of phosphate additives is not quantitatively labeled on the food products [3]. León et al. (2013) analyzed 2394 best-selling branded grocery products for phosphorus content and compared the contents of matched products with and without phosphate additives (using the ingredient list). Phosphate additives were common in prepared frozen foods (72%), dry food mixes (70%), packaged meat (65%), bread and baked goods (57%), soups (54%) and yogurts (51%). The grocery products with phosphate additives contained on average 67 mg/100 g more phosphorus than the matched products without additives [44]. Therefore, calculated intakes based on databases likely underestimate phosphorus intake [3]. To compensate for this, we estimated phosphorus intake from renal phosphorus excretion data according to Morimoto et al. (2014) [17]. They showed that the excretion of phosphorus in 24 h urine can be used to estimate the amount of dietary phosphorus intake and that this method is superior to estimations based on weighed dietary records [17]. Our results showed no difference between the estimated phosphorus intake from renal excretion and weighed dietary records, except for women. For women, phosphorus intake calculated from renal excretion was significantly lower compared to phosphorus intake calculated from weighed dietary record. This can be due to the aspect that in relation to phosphorus intake women excreted less phosphorus via the kidney compared to men (data not shown) and thus, calculated intake (estimated from urine data) is lower. Nevertheless, there was a significant positive correlation between phosphorus intakes estimated from urine data and weighed dietary records. Morimoto et al. (2014) presumed a phosphorus absorption rate of 65% [17]. This rate appears too low for the phosphorus absorption from phosphate additives, but it adequately reflects phosphorus absorption from other sources [25]. A normal mixed diet contains a mixture of added and natural phosphorus. Thus, the absorption rate in the upper range is adequately chosen. One limitation of this method is the increasing fecal excretion of phosphorus due to increasing phosphorus (and calcium) intakes [14,15,42,43]. Morimoto et al. (2014) also emphasized that the differences in phosphorus intake between the two estimations increase when phosphorus intake increases and that phosphorus absorption depends on the balance with calcium intake [17]. Another limitation of this calculation is the influence of renal function, which affects phosphorus excretion and in terms of impaired kidney function leads to an underestimation of phosphorus intake. However, the importance and the advantage of this calculation using urine data is the independence of possible inaccurate databases and weighed protocols. Therefore, it is necessary in further studies to validate such calculations with respect to very high phosphorus intakes and to renal function. In principle, dietary records and 24 h urine collections, together, should be used to assess the phosphorus intake.

Overall limitations of the present investigation are (I) the small sample size and (II) the availability of phosphorus and calcium food sources only from Study 3.

## 5. Conclusions

In conclusion, our data show that there was a constant phosphorus intake in three German study collectives from 2006 to 2014, but that this intake was far above the recommendations. In contrast, calcium intake decreased during the investigation period and was below recommendation in two of three studies. The main sources for phosphorus intake were bread/cereal products, milk/milk products and meat/meat products/sausage products and for calcium milk/milk products/cheese, bread/cereal products as well as beverages. Furthermore, the results of our study indicate that usual phosphorus intakes calculated from phosphorus excretions in urine are comparable to phosphorus intakes estimated by weighed dietary records.

## Figures and Tables

**Table 1:** Overview of study design characteristics.

	Study 1	Study 2	Study 3
Design	Double-blinded, placebo-controlled		
	Cross-over	Parallel	Parallel
Year	2006	2011	2014
Supplement and dosage	Ca_5_(PO_4_)_3_OH, 1 g calcium/day	Ca_5_(PO_4_)_3_OH, 1 g calcium/day	NaH_2_PO_4_, 1 g phosphorus/day
	CaCO_3_, 1 g calcium/day	Vitamin D_3_, 10 µg/day	CaCO_3_, 0.5/1 g calcium/day
	Ca_3_(PO_4_)_2_, 1 g calcium/day		
Duration of each intervention	4 weeks	8 weeks	8 weeks

Ca_5_(PO_4_)_3_OH pentacalcium hydroxy-trisphosphate, NaH_2_PO_4_ monosodium phosphate, CaCO_3_ calcium carbonate.

**Table 2:** Baseline characteristics of the three study collectives.

	Study 1	Study 2	Study 3
n			
All	31	56	62
Women	16	32	30
Men	15	24	32
Age [y]			
All ^W^	24 (2) ^a^	43 (12) ^b^	29 (7) ^c^
Women ^W^	24 (2) ^a^	44 (12) ^b^	28 (7) ^c^
Men ^W^	24 (3) ^a^	41 (13) ^b^	29 (6) ^c^
Body weight [kg]			
All	70 (14)	73 (14)	71(14)
Women	62 (10)	68 (11)	63 (9)
Men	77 (13)	80 (15)	80 (12)
BMI [kg/m^2^]			
All	23 (4)	25 (4)	24 (3)
Women	22 (4)	25 (4)	23 (3)
Men	24 (4)	25 (4)	25 (3)

Data are expressed as mean (standard deviation); ^a,b,c^ mean values within a line with dissimilar superscript letters are significantly different; results without superscript letters are not significantly different; differences between the studies were tested with univariate analysis of variance followed by Bonferroni post hoc test; ^W^ univariate analysis of variance with Welch adjustment was used in case of variance heterogeneity (Tamhame-T2 post hoc test).

**Table 3:** Phosphorus and calcium intakes and serum/plasma concentrations of phosphate and calcium in three studies.

	Study 1	Study 2	Study 3
Phosphorus			
Intake [mg/kg BW]	20 (5)	18 (7)	19 (4)
Plasma/Serum [mmol/L]	^S^ 1.37 (0.16) ^a^	^P^ 1.08 (0.17) ^b^	^P^ 1.21 (0.18) ^c^
Calcium			
Intake [mg/kg BW]	17 (6)	12 (5)	13 (4)
Plasma/Serum [mmol/L] ^W^	^S^ 2.51 (0.18) ^a^	^P^ 2.36 (0.09) ^b^	^P^ 2.34 (0.07) ^b^

*n* = 31 (Study 1); *n* = 56 (Study 2); *n* = 61 (Study 3); data are expressed as mean (standard deviation); BW body weight; ^a,b,c^ mean values within a line with dissimilar superscript letters indicate significant differences; results without superscript letters are not significantly different; differences between the studies were tested with univariate analysis of variance followed by Bonferroni post hoc test; ^W^ univariate analysis of variance with Welch adjustment was used in case of variance heterogeneity (Tamhame-T2 post hoc test); ^S^ concentration in serum; ^P^ concentration in plasma.

**Table 4:** Associations between phosphorus and calcium intake with parameters of phosphorus and calcium status.

	n	All Subjects	Women	Men
Correlations between phosphorus intake and:				
Age	149	−0.119	−0.220	−0.209
Serum/plasma phosphate	149	**0.042** ^S,^*	−0.150 ^P^	0.191 ^S^
Serum/plasma calcium	149	0.000 ^S^	**−0.233** ^S,^*	0.029 ^S^
Calcium intake	**149**	**0.597** ^S,^***	**0.511** ^P,^***	**0.599** ^S,^***
Renal phosphorus	**149**	**0.411** ^S,^***	0.211 ^S^	**0.361** ^S,^**
Renal calcium	**149**	**0.223** ^S,^**	0.052 ^S^	0.152 ^S^
Fecal phosphorus	**79**	**0.219** ^S,^*	**0.303** ^S,^*	0.180 ^S^
Fecal calcium	**79**	**0.234** ^S,^*	**0.364** ^S,^*	0.185 ^S^
Correlations between calcium intake and:				
Age	149	**−0.272** ***	−0.177	**−0.349** **
Serum/plasma phosphate	149	0.122 ^S^	0.126 ^P^	0.071 ^S^
Serum/plasma calcium	149	0.105 ^S^	−0.061 ^S^	0.192 ^S^
Phosphorus intake	**149**	**0.597** ^S,^***	**0.511** ^P,^***	**0.599** ^S,^**
Renal phosphorus	**149**	**0.198** ^S,^*	0.170 ^S^	0.158 ^S^
Renal calcium	**149**	**0.237** ^S,^**	0.177 ^S^	**0.274** ^S,^*
Fecal phosphorus	**79**	**0.442** ^S,^**	**0.480** ^S,^***	**0.441** ^S,^**
Fecal calcium	**79**	**0.563** ^S,^***	**0.559** ^S,^***	**0.545** ^S,^**

Expressed are correlation coefficients; ^S^ Spearman correlation coefficient due to at least one not normally distributed data group; ^P^ Pearson correlation coefficient due to normally distributed data groups; * correlation coefficient *p* ≤ 0.05; ** correlation coefficient *p* ≤ 0.01; *** correlation coefficient *p* ≤ 0.001; bold coefficients indicate significance.

**Table 5:** Contribution of different food product groups to phosphorus and calcium intake.

	Phosphorus [%]	Calcium [%]
**Breads/cereal products (baked goods)**	**30.7**	**12.1**
Bread	17.5	6.1
Dishes based on bread	0	0
Baked goods	5.2	3.7
Cereals/cereals products	6.7	1.4
Dishes based on cereals/cereals products	1.3	0.9
**Vegetables/mushrooms/legumes**	**4.5**	**5.9**
Vegetables	3.4	4.4
Vegetables products	0.2	0.2
Mushrooms	0	0
Legumes	0.5	0.6
Dishes based on vegetables	0.5	0.6
**Potatoes/potato products**	**2.4**	**1.4**
Potatoes/potato products	2.1	1.2
Other tubers	0	0
Dishes based on potatoes	0.3	0.2
**Fruit/fruit products**	**1.4**	**2.0**
Fruits	1.2	1.4
Fruits products	0.2	0.5
Dried fruits	0	0
**Nuts/seeds**	**1.0**	**0.3**
**Fats**	**0.2**	**0.2**
Animal fats	0.2	0.2
Vegetable fats	0	0
**Milk/milk products/cheese**	**23.3**	**46.0**
Milk and milk beverages	6.8	13.0
Milk products	2.7	6.1
Cheese and curd	12.9	24.8
Dishes based on milk/milk products	0.9	2.2
**Eggs**	**3.7**	**1.6**
Eggs	0.1	0.0
Dishes based on eggs	3.6	1.6
**Meat/meat products/sausage products**	**11.7**	**1.9**
Meat	3.9	0.5
Meat and sausage products	4.7	0.8
Dishes based on meat	3.2	0.6
**Fish/fish products/crustaceans**	**3.6**	**0.7**
Fish and fish products	3.1	0.5
Crust and shellfish	0.2	0.1
Dishes based on fish/crustaceans	0.3	0
**Soup/stew**	**1.8**	**1.6**
**Sauce/flavor-giving ingredients**	**1.2**	**0.9**
Sauces	0.9	0.6
Flavor-giving ingredients	0.2	0.3
**Sweets**	**5.3**	**7.5**
Sweets	4.1	5.9
Ice cream	1.0	1.5
Sweet spreads	0.2	0.1
Sweeteners	0	0
**Snacks**	**0.8**	**0.4**
Snacks based on potatoes	0.2	0.1
Nuts and mixed nuts (roasted/salted)	0	0
Peanut flips/popcorn	0.2	0
Salty biscuits	0.1	0.2
**Beverages**	**6.8**	**16.2**
Water	0	7.6
Coffee and Tea	1.4	4.2
Fruit tea and herb tea	0	0
Fruit juice or nectar	1.3	2.3
Vegetable juice	0	0
Soft drinks	1.3	0.7
Other non-alcoholic	0	0
Beer	3.3	0.8
Wine and sparkling wine	0.4	0.5
Liquor	0	0
Other alcoholic	0.1	0.2
**Other**	**1.8**	**1.1**

**Table 6:** Comparison of phosphorus intake calculated from weighed dietary records with estimations of phosphorus intake from renal phosphorus excretion.

Calculated Phosphorus Intakes from:	Study 1	Study 2	Study 3	All Studies
Weighed dietary records [mg/day]	1364 (382)	1307 (443)	1353 (328)	1338 (384)
Phosphorus excretion in urine [mg/day] ^1^	1344 (505)	1203 (426)	1306 (477)	1275 (465)

*n* = 31 (Study 1); *n* = 56 (Study 2); *n* = 61 (Study 3); data are expressed as mean (standard deviation); results without superscript letters are not significantly different; ^1^ phosphorus intake estimated from renal phosphorus excretion according to Morimoto et al. (2014) [17].

## Supplementary Materials

The following are available online at http://www.mdpi.com/2072-6643/10/2/171/s1, Table S1: Product group food item description and examples according to the “National Consumption Study II”, Table S2: Baseline phosphorus and calcium parameters of women and men from three studies.
