# Supplementary material for: Habitual Intakes, Food Sources and Excretions of Phosphorus and Calcium in Three German Study Collectives
Source: Nutrients. 2018 Feb 2;10(2):171. doi: 10.3390/nu10020171 (PMC5852747; doi:10.3390/nu10020171)
Supplement: Supplementary file 1 [file nutrients-10-00171-s001.pdf]

Supplementary Materials:

Table S1 Product group food item description and examples according to the "National Consumption Study II" [16].

Product group	Description and example food items
Bread/cereal products (baked goods)	
Bread	whole meal bread, multigrain bread, brown bread, white bread, crispbread, whole meal roll, multigrain roll, white roll, rye roll, pretzel roll, cornbread, rice bread, rusk
Dishes based on bread	proven rolls, club sandwich with tuna, toast "Hawaii", serviette dumpling
Baked goods	cakes and tortes, baked goods spicy (pizza, onion cake) and sweet
Cereals/cereal products	cereals and flours, rice (processed), muesli (dry), pasta products, bulgur
Dishes based on cereals/cereal products	pasta dishes, rice dishes, patties
**Vegetables, mushrooms and legumes**	
Vegetables	raw vegetables (green salad, tomato), cooked vegetables (broccoli, carrot)
Vegetable products	gherkin, sour preserves, olives, corn preserve
Mushrooms	steamed chanterelle, steamed mushroom
Legumes	roasted soybean, kidney bean preserve
Dishes based on vegetables	salad (cooked vegetables and raw vegetables)
**Potatoes and potato products**	
Potatoes/potato products	cooked potatoes with salt, mashed potatoes, potato pancakes, potato dumplings
Other tubers	sweet potato, topinambur
Dishes based on potatoes	potato salad, potato gratin
**Fruit and fruit products**	
Fruits	unprocessed fruits
Fruit products	sugared and heated fruits
Dried fruits	dried apple, dried peach
**Nuts and seeds**	cashew nuts, peanuts
**Fats**	
Animal fats	herb butter, cultured butter, butter with yoghurt

Vegetable fats	half-fat margarine, margarine with olive oil
Milk, milk products and cheese	
Milk and milk beverages	cow's milk 3.5% fat, cocoa, milkshake
Milk products	kefir, buttermilk, sour milk, whey, yoghurt, yoghurt with flavor-giving ingredients (milk and yoghurt: full-fat, reduced-fat, no information) cream, sour cream, coffee cream
Cheese and curd	hard cheese, soft cheese, cream cheese and curd or junket (some cheeses: full-fat, reduced-fat, no information) sour cream-, cooked-, whey- and processed cheese
Dishes based on milk/milk products	rice pudding, cornflakes (prepared), cheese dishes
**Eggs**	
Eggs	scrambled egg, fried egg, boiled egg
Dishes based on eggs	pancakes, egg salad, egg in mustard sauce
Meat, meat products and sausage products	
Meat	beef, veal, pork, poultry, meat from other animals, minced meat, innards
Meat and sausage products	sausage, smoked pork, salami, krakauer, smoked meat, ham
Dishes based on meat	meatball, pork schnitzel, goulash
Fish, fish products and crustaceans	
Fish and fish products	baked filet, smoked trout
Crust and shellfish	crab preserve, grilled king prawns, mussels fresh cooked
Dishes based on fish/crustaceans	fish fingers, mussels in white wine sauce
**Soup and stew**	noodle soup with chicken, potato soup with sausage
Sauce and flavor-giving ingredients	
Sauces	warm sauces, cold sauces (also fruit sauces and ketchup)
Flavor-giving ingredients	mustard, vinegar
**Sweets**	
Sweets	chocolate, other products from chocolate, sweets and candies, muesli bars, fruit bars
Ice cream	ice cream, soft ice cream
Sweet spreads	fruit spread, jam, honey, syrup, chocolate spread, nut spread
Sweeteners	sugar, sugar substitute, sweetener

**Snacks**	
Snacks based on potatoes	chips
Nuts and mixed nuts (roasted/salted)	roasted peanuts, roasted almonds sweet and salted, roasted and salted sunflower seeds
Peanut flips/popcorn	puffed rice, popcorn
Salty biscuits	tortilla chips, pretzel sticks
**Beverages**	
Water	mineral water, drinking water
Coffee and tea	cappuccino, coffee, green tea, black tea
Fruit tea and herb tea	peppermint tea, mate tea, rooibos tea
Fruit juice or nectar	apple fizz, orange fizz, multivitamin juice
Soft drinks	orangeade, cola
Fruit drinks	ACE fruit juice, wellness drink
Other non-alcoholic	malt beer, sparkling wine alcohol-free
Beer	dark beer, pilsner
Wine and sparkling wine	red wine, with wine, mulled wine
Liquor	whiskey, egg liqueur, grappa
Other alcoholic	punch, fizz
**Other**	soya drinks, tofu, meat and sausage substitute from soya, cereal spreads, vegetarian lard, vanilla cream, nut cream

Table S2 Baseline phosphorus and calcium parameters of women and men from three studies.

	Study 1		Study 2		Study 3	
	Women (n = 15)	Men (n = 16)	Women (n = 32)	Men (n = 24)	Women (n = 30)	Men (n = 32)
Phosphorus						
Intake [mg/d][1]	1177 (316)	1539 (362)	1194 (318)	1457 (539)	1187+ (268)	1530 (294)
Intake [mg/kg BW]	19 (5)	21 (6)	18 (6)	19 (7)	19 (5)	20 (4)
Estimated intake[2] [mg/d]	1013 (320)	1654 (449)	1057 (306)	1397 (489)	1017+ (299)	1614 (438)
Serum/plasma phosphate [mmol/l]	[S]1.38 (0.14) [a]	[S]1.35 (0.18) [a]	[P]1.06 (0.12) [b]	[P]1.10 (0.21) [b]	[P]1.20 (0.17) [c]	[P]1.22 (0.19) [ab]
Urine [mg/d]	658 (208)	1075 (292)	687 (202)	908 (318)	661 (194)	1049(285)
Faeces [mg/d]	665 (252)	736 (359)	529 (241)	533 (280)	-	-
Calcium						
Intake [mg/d]	1147 (372)	1153 (430)	840 (252)	896 (398)	819 (239)	977 (321)
Intake [mg/kg BW]	19 (5) [a]	15 (6)	13 (4) [b]	12 (6)	13 (4) [b]	13 (4)
Serum/plasma [mmol/l]	[S]2.59 (0.23) [a]	[S]2.44 (0.07) [a]	[P]2.35 (0.09) [b]	[P]2.38 (0.08) [b]	[P]2.31 (0.05) [b]	[P]2.37 (0.07) [b]
Urine [mg/d]	114 (51)	222 (81) [a]	133 (64)	150 (80) [b]	125 (49)	155 (68) [b]
Faeces [mg/d]	883 (270)	1027 (510) [a]	749 (360)	687 (324) [b]	-	-

Data are expressed as mean (standard deviation); BW body weight; [a, b] mean values within a line within a gender with dissimilar superscript letters are significantly different; results without superscript letters are not significantly different; differences between the studies were tested with univariate analysis of variance followed by Bonferroni post hoc test; + similar symbols indicate significance ($p = 0.004$), tested with Student´s t test; [S] concentration in serum; [P] concentration in plasma; [1] phosphorus intake calculated from weighed dietary record; [2] phosphorus intake estimated from renal phosphorus excretion according to Morimoto et al. (2014) [17].
